# The Triage Capability of Laypersons: Retrospective Exploratory Analysis

**DOI:** 10.2196/38977

**Published:** 2022-10-12

**Authors:** Marvin Kopka, Markus A Feufel, Felix Balzer, Malte L Schmieding

**Affiliations:** 1 Institute of Medical Informatics Charité – Universitätsmedizin Berlin, corporate member of Freie Universität Berlin and Humboldt-Universität zu Berlin Berlin Germany; 2 Cognitive Psychology and Ergonomics Department of Psychology and Ergonomics (IPA) Technische Universität Berlin Berlin Germany; 3 Division of Ergonomics Department of Psychology and Ergonomics (IPA) Technische Universität Berlin Berlin Germany

**Keywords:** digital health, triage, self-triage, urgency assessment, patient-centered care, care navigation, decision support, symptom checker, care, support, medical, health professional, patient, self-assessment, decision, accuracy, error, sensitivity, emergency, female, male

## Abstract

**Background:**

Although medical decision-making may be thought of as a task involving health professionals, many decisions, including critical health–related decisions are made by laypersons alone. Specifically, as the first step to most care episodes, it is the patient who determines whether and where to seek health care (triage). Overcautious self-assessments (ie, overtriaging) may lead to overutilization of health care facilities and overcrowded emergency departments, whereas imprudent decisions (ie, undertriaging) constitute a risk to the patient’s health. Recently, patient-facing decision support systems, commonly known as symptom checkers, have been developed to assist laypersons in these decisions.

**Objective:**

The purpose of this study is to identify factors influencing laypersons’ ability to self-triage and their risk averseness in self-triage decisions.

**Methods:**

We analyzed publicly available data on 91 laypersons appraising 45 short fictitious patient descriptions (case vignettes; N=4095 appraisals). Using signal detection theory and descriptive and inferential statistics, we explored whether the type of medical decision laypersons face, their confidence in their decision, and sociodemographic factors influence their triage accuracy and the type of errors they make. We distinguished between 2 decisions: whether emergency care was required (decision 1) and whether self-care was sufficient (decision 2).

**Results:**

The accuracy of detecting emergencies (decision 1) was higher (mean 82.2%, SD 5.9%) than that of deciding whether any type of medical care is required (decision 2, mean 75.9%, SD 5.25%; *t*_>90_=8.4; *P*<.001; Cohen *d*=0.9). Sensitivity for decision 1 was lower (mean 67.5%, SD 16.4%) than its specificity (mean 89.6%, SD 8.6%) whereas sensitivity for decision 2 was higher (mean 90.5%, SD 8.3%) than its specificity (mean 46.7%, SD 15.95%). Female participants were more risk averse and overtriaged more often than male participants, but age and level of education showed no association with participants’ risk averseness. Participants’ triage accuracy was higher when they were certain about their appraisal (2114/3381, 62.5%) than when being uncertain (378/714, 52.9%). However, most errors occurred when participants were certain of their decision (1267/1603, 79%). Participants were more commonly certain of their overtriage errors (mean 80.9%, SD 23.8%) than their undertriage errors (mean 72.5%, SD 30.9%; *t*_>89_=3.7; *P*<.001; *d*=0.39).

**Conclusions:**

Our study suggests that laypersons are overcautious in deciding whether they require medical care at all, but they miss identifying a considerable portion of emergencies. Our results further indicate that women are more risk averse than men in both types of decisions. Layperson participants made most triage errors when they were certain of their own appraisal. Thus, they might not follow or even seek advice (eg, from symptom checkers) in most instances where advice would be useful.

## Introduction

### Background

Increased emergency department (ED) crowding and longer waiting times are associated with higher mortality [1,2], increased adverse events [3], and worse patient outcomes in general [4]. To mitigate these problems, each patient’s urgency is assessed upon arrival in the ED—a process called triage. Usually, triage decisions are made by nurses and trained specialists, whose workload is generally high [5]. Increased workload and overcrowding pose huge risks as triage errors currently occur in roughly 16% of all cases [6] and nurses often rely on their intuition and speed up triage by not collecting further information, for example, medical history and physiological data [7,8]. These risks and the workload for health care workers could be reduced if patients were guided to appropriate health care facilities before visiting an ED to receive an initial assessment.

Getting patients to visit the health care facility most suitable for their symptoms can, for example, be achieved by offering phone triage, a telephone hotline patients can call to get a remote urgency assessment of their symptoms. For example, Roivainen et al [9] found that giving medical advice was sufficient for one-third of callers with nonemergent cases, and ED workload subsequently decreased by 36%. Another study by Midtbø et al [10] found that ED attendance decreased from 68.7% to 23.4% when advertising telephone triage hotlines. However, the accuracy of triage hotlines seems to be only around 71% with undertriaging occurring in 12% of all cases [11]—which could be potentially dangerous for some patients. Furthermore, a study based on commercial claims data from 2011 to 2013 concludes that direct-to-consumer telehealth may increase health care utilization and costs by making access to health care more convenient. Thus, such services may shift and increase, rather than reduce, the demand for health care services [12].

Another solution to disburden health care services lies in empowering patients to self-assess their medical complaints and thereby improving their ability to adequately decide which type of health care facility to visit (ie, self-triage), or where appropriate to stay at home and care for themselves. Since these decisions are made by laypersons instead of medical professionals, they come with various challenges. For example, previous studies indicate that laypersons tend to overtriage [13] and that women rate symptoms as more urgent than men [14] and are thus potentially even more inclined toward overtriage. Moreover, an Australian study showed that although laypersons are risk averse when making triage decisions, they cannot reliably detect emergencies either [15].

To address these deficits, decision support systems are designed to aid laypersons in their self-triage decision-making process, for example, kiosks in the ED [13] or symptom checkers [16]. When using them, around 25% of all patients seem to have reduced perceived urgency of their complaints, and in an experimental study, most participants also followed the advice received by the symptom checkers [17,18]. However, the accuracy of these systems varies widely [19-22]. Implementing such decision aids raises several new questions: can laypersons adequately judge when to seek such decision support? Would all laypersons profit the same way from such decision support tools? Which are the most challenging decisions to laypersons, that is, where is advice from a decision support tool most needed?

### Objective

To provide a foundation for tackling these questions, in this study we investigate whether laypersons’ triage accuracy and their risk averseness differ by sociodemographic variables and whether laypersons can potentially gauge whether they require advice in their decision-making, by exploring if laypersons’ confidence in their triage appraisals functions as a reliable predictor of accuracy.

## Methods

### Ethical Considerations

No ethics approval was required for this study. Approval of the original study [23] was granted by the Ethics Committee of the Department of Psychology and Ergonomics (IPA) at Technische Universität Berlin (Tracking number: FEU_03_20180615). Participants volunteered to participate in the survey, and informed consent was obtained.

### Data Collection

This analysis builds on data collected in a previous study by Schmieding et al [23], which was made available in a public open data repository [24]. They compared laypersons’ triage capabilities with symptom checker performance, which are tools developed to provide clinical decision support to laypersons. Their study found that laypersons’ overall triage accuracy was mediocre (mean 60.9%, SD 6.8%) based on a set of 45 fictitious patient descriptions (case vignettes). These 45 case vignettes were originally compiled by Semigran et al [21], whose study reported a mediocre overall triage accuracy for a sample of 15 symptom checkers, which was similar to laypersons’ accuracy in Schmieding et al’s study [23].

The layperson sample consisted of 91 US residents without prior professional medical training. They were recruited from Amazon Mechanical Turk (MTurk) in March 2020. Participants were paid US $4 for completing the web-based survey and assessing the 45 case vignettes unaided. As an incentive, a bonus of US $3 was rewarded if they achieved an accuracy above 58%. Compared with the US general population, the layperson sample had a higher level of education (all participants had at least a high school degree) and included a higher proportion of men (55/91, 60.4%) than women (36/91, 39.5%).

A more detailed description of the participants’ characteristics is provided by Schmieding et al [23]. Here, we use their data set to further explore individual differences influencing laypersons’ triage assessment and decisions.

### Web-Based Survey

Schmieding et al [23] developed a web-based survey in which participants were asked to rate the urgency of presented case vignettes. They adapted 45 case vignettes from Semigran et al [21], comprising 15 cases for each of the 3 triage levels (self-care, nonemergency, or emergency care). The vignettes, as chosen by Semigran et al [21], included both common and uncommon chief complaints from a wide range of diagnoses and were collected from various clinical sources, including teaching materials for health care professionals.

After every triage assessment, participants were asked how certain they were in their assessment on a 4-point Likert-scale (“Very uncertain,” “Rather uncertain,” “Rather certain,” and “Very certain”). Three sociodemographic variables were surveyed (gender, age, and level of education) and rated on a 5-tiered ordinal scale (“Non-high school graduate,” “High school graduate,” “Some college,” “Bachelor’s degree,” and “Graduate degree”).

### Data Analysis

We conducted analyses and generated the images using base R 4.0.5 (R Core Team) [25] and the packages ggplot2 [26], RColorBrewer [27], and tidyverse packages [28].

We dichotomized triage levels to explore whether laypersons could reliably distinguish whether emergency care was required or not (decision 1), and whether self-care was sufficient or not (decision 2). Whether and where health care should be sought are the 2 common questions symptom checkers are approached with [19,29]. For decision 1, we grouped self-care cases with nonemergency cases to assess whether participants were able to correctly detect when emergency care is necessary. For decision 2, we combined emergency and nonemergency cases to the category “health care” to verify whether participants were able to correctly assess when seeing a health care professional rather than staying at home is appropriate. For each of these binary decisions, we calculated means and standard deviations for common metrics of signal detection theory (accuracy, sensitivity, specificity, and negative and positive predictive values). When determining negative and positive predictive values (NPVs and PPVs), we used the occurrence rate within the sample of case vignettes as prevalence.

To explore which factors influence laypersons’ risk averseness, triage accuracy, and confidence in their own triage appraisal, we used linear models to quantify relationships between continuous variables (age and risk averseness) and compared proportions between subgroups for ordinal and categorical variables (gender, education, and certainty).

Overtriage errors were defined as appraising the case’s urgency as more urgent than necessary (eg, the participant suggested emergency care when nonemergency care was appropriate) and undertriage errors as judging it as less urgent than required. Risk averseness was defined as the proportion of overtriage errors compared to all errors, whereas a participant’s triage accuracy was calculated as the proportion of correctly solved cases related to all cases.

## Results

### Laypersons’ Triage Capability in Binary Triage Levels

**Figure 1:**
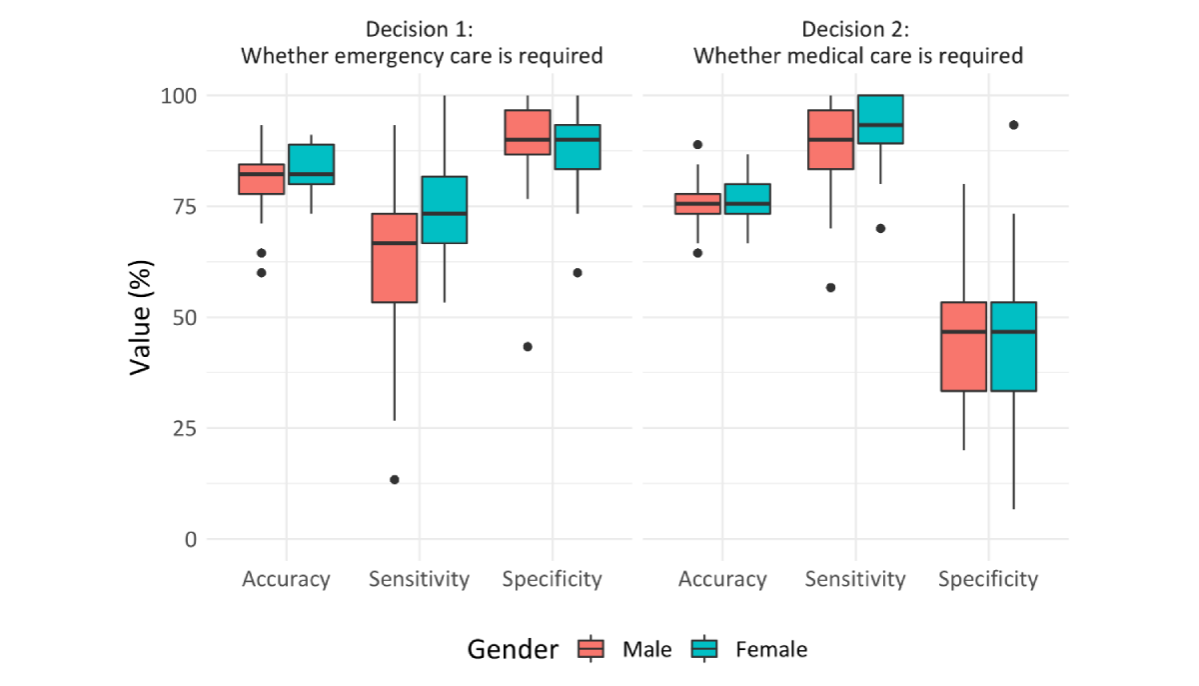
Accuracy, sensitivity, and specificity for 2 binary triage decisions by participants’ gender.

On average, the participants were able to correctly classify whether a fictitious patient required emergency care or not in about 36 out of 45 cases (mean 82.2%, SD 5.88%; see decision 1 in Figure 1). The majority of participants (85/91, 93.4%) achieved an accuracy of 75% or higher. Sensitivity for detecting emergencies (mean 67.5%, SD 16.4%) was lower than the corresponding specificity (mean 89.6%, SD 8.6%), that is, the rate of assessments where cases not requiring emergency care were correctly classified as such. The PPV for detecting emergencies (mean 78.5%, SD 11.8%) was lower than the NPV (mean 85.3%, SD 6.0%).

Concerning decision 2 (Figure 1) on whether or not professional medical care is required, the overall accuracy was lower on average (mean 75.9%, SD 5.25%). Two-thirds of participants (58/91, 63.7%) achieved an accuracy of 75% or greater concerning decision 2. Here the observed sensitivity (mean 90.5%, SD 8.3%) was higher than the corresponding specificity (mean 46.7%, SD 15.9%). The PPV for this decision (mean 77.6%, SD 4.7%) was similar to the NPV (mean 76.1%, SD 16.5%).

The difference in accuracy between decision 1 and decision 2 was found to be statistically significant in a post-hoc 2-sided *t* test (*t*_>90_=8.44; *P*<.001) with a large effect size (Cohen *d*=0.88). The described patterns are similar when values for accuracy, sensitivity, and specificity are broken down by gender (Figure 1). However, male participants missed a case that required medical care 1.4 times more often than female participants (false negative rates for decision 2 of 10.8%, 178/1650, for men and 7.6%, 82/180, for women), and 1.5 times more often that a case required emergency care (false negative rates for decision 1 of 37.8%, 312/825, for men and 24.4%,132/540, for women).

### Demographic Variables and Laypersons’ Risk Averseness

Participants were generally risk averse [23]. Most of their errors were overtriage errors. Only a small portion of participants (24/91, 27%) made more undertriage errors than overtriage errors.

**Figure 2:**
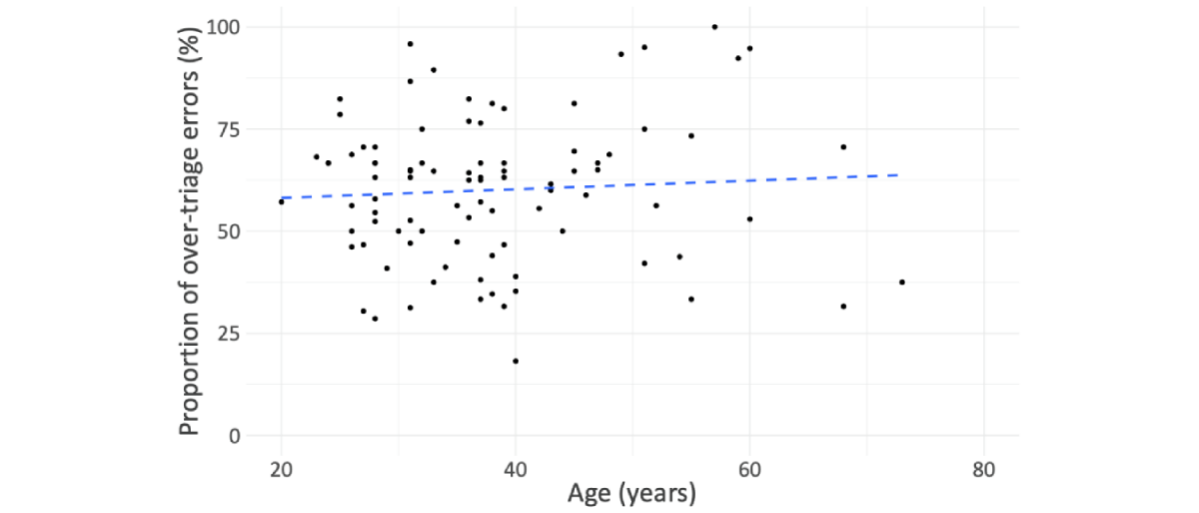
Risk averseness by age.

**Figure 3:**
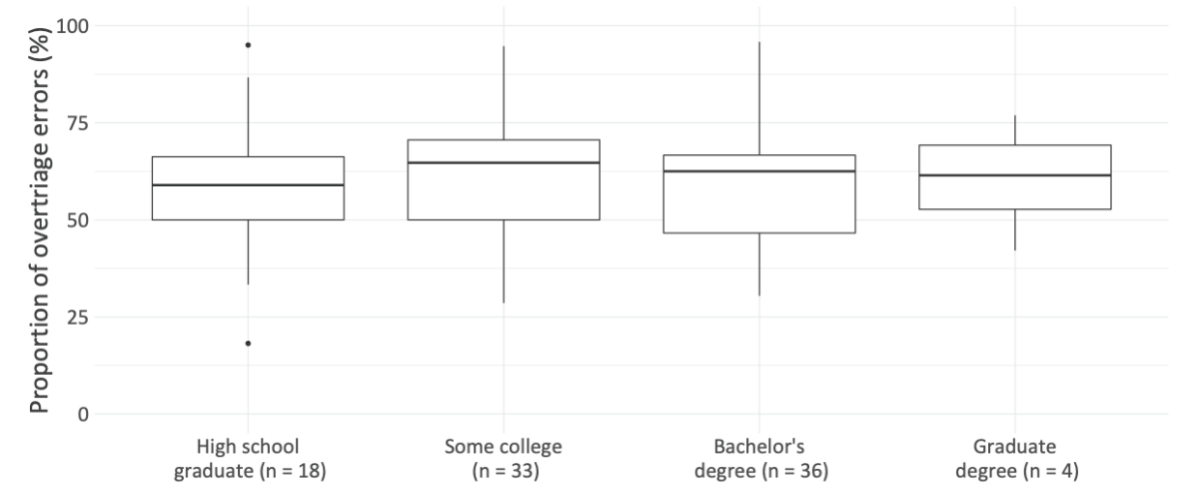
Risk averseness by education.

**Figure 4:**
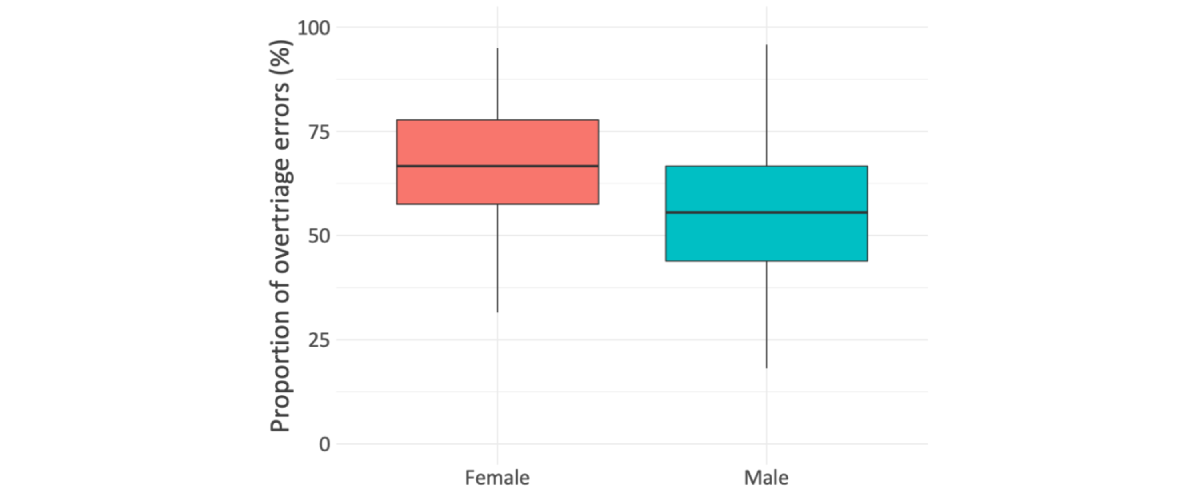
Risk averseness by gender.

Age only explained little variance in the decisions made (*R*^2^=0.004; Figure 2), and median risk averseness was similar for each education level (Figure 3). Risk averseness varied with gender; that is, female participants were more risk averse than their male counterparts (Figure 4): for male participants, the ratio of overtriage to undertriage error was 1.2:1 (549:452 vignette evaluations), in contrast to 2:1 for female participants (407:195 vignette evaluations). Subsequently, the average female participant’s proportion of overtriage errors among all errors was higher (mean 65.9%, SD 17.3%) than the respective proportion for male participants (mean 55.6%, SD 16.0%). This difference was found to be statistically significant in a post-hoc 2-sided Welch *t* test (*t*_>68.6_=2.85; *P*=0.006) with a medium effect size (*d*=0.62).

### Decisional Certainty and Laypersons’ Triage Behavior

#### Overall Decisional Certainty

Participants were certain in most of their triage appraisals. They indicated being “very certain” or “rather certain” in about 4 out of 5 (3381/4095, 82.6%) of the triage assessments, see Table 1 for details. Only a small portion of participants (33/91, 36.2%) indicated having been “very uncertain” in one or more of their triage decisions.

**Table 1:** Triage assessment and accuracy by certainty.

Triage assessment	Degree of certainty				Total
	“Very uncertain”	“Rather uncertain”	“Rather certain”	“Very certain”	
Correct	24	354	1145	969	2492
Incorrect	34	302	886	381	1603
Total	58	656	2031	1350	4095
Accuracy (%)	41.4	53.9	56.3	71.8	60.8

#### Decisional Certainty and Triage Accuracy

Participants’ triage accuracy varied with their degree of certainty, see Table 1: it was the highest when they indicated being “very certain” (969/1350, 71.8%) and the lowest when they indicated being “very uncertain” (24/58, 41.4%). For decisions where participants were uncertain (pooling “rather uncertain” and “very uncertain”), the accuracy of their triage decision was lower (378/714, 52.9%) than when being rather or very certain (2114/3381, 62.5%; see Table 2).

When mistaken, participants were on average still commonly certain about their assessment, though less certain than when their appraisal was correct (see Tables 1 and 2). When being correct, they were certain in 84.9% (2114/2492) of the correct assessments, whereas they were certain in 79.0% (1267/1603) of all incorrect assessments. This difference is statistically significant in a post-hoc paired-sample *t* test (*t*_>90_=5.43; *P*<.001; *d*=0.57).

**Table 2:** Triage assessment and accuracy by dichotomized certainty levels.

Degree of certainty	Triage assessment		Total	Accuracy (%)
	Correct	Incorrect		
Uncertain	378	336	714	52.9
Certain	2114	1267	3381	62.5
Total	3381	1603	4095	60.8
Proportion certain (%)	87	79	N/A^a^	N/A

^a^N/A: not applicable.

#### Decisional Certainty and Type of Error

Regarding the 2 types of errors, that is, overtriaging and undertriaging, we observed that participants’ decisional certainty was higher when overtriaging (proportion of overtriage errors where they were either rather or very certain, 783/956, 81.9%) than when undertriaging (484/647, 74.8%). The ratio of overtriage to undertriage errors increased with the level of certainty, from 1.12:1 (18:16) for appraisals where participants were very uncertain to 2:1 (254:127) for appraisals where participants were very certain, see Table 3. The average proportion of participants being certain while overtriaging was higher (mean 80.9%, SD 23.8%) than that while undertriaging (mean 72.5%, SD 30.9%). This difference was statistically significant in a post-hoc paired-sample *t* test (*t*_>89_=3.70; *P*<.001) with a small effect size (*d*=0.39).

**Table 3:** Triage errors by certainty.

Error type	Degree of certainty, n (%)				Total
	“Very uncertain”	“Rather uncertain”	“Rather certain”	“Very certain”	
Overtriage	18 (1.9)	155 (16.2)	529 (55.3)	254 (26.6)	956
Undertriage	16 (2.5)	147 (22.7)	357 (55.2)	127 (19.6)	647
Total	34 (2.1)	302 (18.8)	886 (55.3)	381 (23.8)	1603

## Discussion

### Principal Findings

As previously reported [15,23], laypersons’ ability to triage is systematically biased toward overtriage, but they tend to miss emergency cases. However, when analyzing actionable metrics of triage ability, we see a majority of laypersons being fairly competent (accuracy ≥75%) in deciding whether they should seek health care (decision 2) and whether emergency care is required (decision 1). The analysis of these binary decisions also revealed a more differentiated insight into laypersons’ risk averseness: they seem to be risk averse regarding decision 2 and thus tend to seek care unnecessarily, but not regarding decision 1, which suggests that they have problems identifying emergencies.

Concerning decision 1, participants were more prone to undertriage (ie, not identifying emergencies) and made only a few overtriage errors. In decision 2, they mostly made overtriage errors (ie, not recognizing that self-care is sufficient) and only a few undertriage errors. This adds further evidence to previous findings that laypersons tend to overtriage [13], but it extends them and shows that this is true only when deciding between self-care and need for a health care professional. Indeed, laypersons were more likely to undertriage when looking at emergency cases. This supports results from a study by Mills et al [15], who found that laypersons often do not recognize emergencies. It also suggests that even when assuming symptom checkers will become highly reliable and patients would use them to improve their decision-making, the benefit of these decision aids might help to disburden health care facilities of low-acuity care but not emergency care; the number of patients (rightly) presenting to the ED might increase with decision aids.

Our results indicate that women are more risk averse and overtriage more than men. This finding is in line with a previous study that found women to rate their symptoms as more urgent [14]. The two other demographic variables we examined (level of education and age) were not associated with accuracy and risk averseness.

As current symptom checkers used to assist laypersons in their triage decisions are rather risk averse [21,30], they would therefore be of greater benefit to men, who made more unsafe decisions. However, women appear to be more regular users of symptom checkers [29].

Participants’ judgement of their decisional certainty predicted to some extent whether their stand-alone triage assessment was correct: when uncertain, participants were more likely to make incorrect assessments than when being certain. At first glance, this suggests that perceived uncertainty is a good prompt for when to seek decision support. However, whether correct or incorrect, our study participants were certain about most of their judgements. Thus, perceived certainty is not a reliable predictor of triage errors, and based on their level of certainty, it seems likely that laypersons may not be able to correctly determine when they would benefit from a decision aid. Especially as participants’ certainty was greater when overtriaging than when undertriaging, even perfect decision aids might not effectively reduce unnecessary doctor’s visits and disburden health care facilities. This contrasts with the suggestions by Winn et al [17]. Their study found that users’ perceived urgency to seek health care commonly decreased after encountering a symptom checker. Their study, however, does not consider whether the provided advice was correct. Therefore, it remains an open question, whether the use of symptom checkers (even when assuming perfect accuracy) can contribute to disburdening health care facilities.

### Limitations

Limitations regarding participants and the evaluation of symptom checker accuracy are reported in detail by Schmieding et al [23] and Semigran et al [21]. Here we report the most important limitations again. Among the main limitations are that the sample of case vignettes is neither exhaustive (eg, mental health issues were excluded) nor proportionate to the incidence of diseases or medical complaints in a real-world setting. Thus, in particular, the reported values for NPVs and PPVs are not to be taken at face value, because they only reflect the prevalence in the sample of vignettes.

Second, although it has been reported that case vignettes are a valid method to assess the health care decision-making of physicians [31-33], the external validity of case vignette–based approaches with layperson decision makers has not been explored yet; that is, laypersons may decide very differently when assessing clinical vignettes compared to when they assess their own or someone else’s medical complaints in the real world [21,23].

Beyond that, there are further limitations specific to the analyses in this study. Our results may have limited external validity: first, the sample was not representative of the US general population; second, triage appraisals might be influenced by the context of the health care system and by recruiting participants online; and third, population groups with no or low (information) technology affinity are not represented. Thus, future studies with more representative panels are required to determine whether our findings hold true for the broader population and whether factors other than gender and perceived certainty influence laypersons’ health care decisions (eg, eHealth literacy [34], health anxiety or hypochondria [35], or propensity to trust [36]). In turn, this knowledge may help to specify for which decision and for whom decision aids would provide a benefit [34-36].

We applied statistical significance testing sparingly, since this study is a retrospective exploratory analysis and was primarily intended to generate hypotheses that can be tested (experimentally) in future studies to draw inferences.

Methodologically, we considered certainty as a measure of whether participants would consult decision aids and whether they would be open to incorporating the recommendations into their decision-making. This implies that perceived certainty in one’s own appraisal correlates inversely with the openness to follow contradicting advice. However, this relationship is not based on any data. Future studies need to test this assumption.

### Conclusions

Our study suggests that laypersons are overcautious in deciding whether they require medical care (decision 2). At the same time, they miss identifying a considerable proportion of emergencies (decision 1). Our results also indicate that women are more risk averse than men in both these decisions. When providing correct advice, decision aids such as symptom checkers could be of benefit to users as they could help reduce the number of missed emergencies and unnecessary visits to low-acuity care facilities. Thus, from a health system’s perspective, decision aids might disburden health care facilities more of low-acuity care than of emergency care. However, layperson participants made most triage errors, and especially overtriage errors, when being certain of their own appraisal. Thus, they might not follow or even seek such advice in most instances where advice would be useful. More studies are needed to better understand laypersons’ ability to self-triage, how this ability could be improved, how decision aids may support laypersons’ medical decision-making, and when laypersons are willing to take advice from decision aids.

## Abbreviations

ED: emergency department
NPV: negative predictive value
PPV: positive predictive value
